# Isolation, identification, and biological control in vitro of tail rot pathogen strain from *Hippocampus kuda*

**DOI:** 10.1371/journal.pone.0232162

**Published:** 2020-04-24

**Authors:** Fangyan Jiang, Hai Huang, Ning Yang, Huimin Feng, Yu Li, Bingbing Han

**Affiliations:** 1 Key Laboratory of Utilization and Conservation for Tropical Marine Bioresources of Ministry of Education, Hainan Tropical Ocean University, Sanya, China; 2 Key Laboratory of Tropical Marine Fishery Resources Protection and Utilization of Hainan Province, Hainan Tropical Ocean University, Sanya China; 3 Sanya Science & Technology Academy of Hainan National Breeding and Multiplication, Sanya, China; Tallinn University of Technology, ESTONIA

## Abstract

Tail rot disease is associated with major economic losses in the seahorse aquaculture in China. This study aimed to isolate and identify the pathogen causing tail rot disease in seahorses. Three culturable intestinal bacteria strains were isolated from *Hippocampus kuda* specimens with tail rot disease. Strain HL11, HL12, and HL13 were identified as *Pseudoalteromonas spongiae*, *Bacillus subtilis* and *Photobacterium ganghwense* based on its morphological characteristics, physiological and biochemical properties, through 16S rRNA and *gyrB* sequencing, respectively. Challenge experiments using these strains on healthy *H*. *kuda* and bacterial re-isolation from challenged diseased seahorses showed that the bacteria strain named HL11 induced identical pathological symptoms, indicating that it is the causative pathogen of the disease. Antibiotic-resistance tests against of 32 antibiotics revealed that HL11 was highly sensitive to 13 kinds, while exhibited intermediate susceptibility to 6, and resistance to 13 kinds. Antibacterial tests of the bioactive agents showed that HL11 was susceptible to five kinds, including tea polyphenols, lactic acid, gallic acid, allicin, and polylysine; however, it was not susceptible to the other 13 kinds of bioactive agents. The results demonstrate the potential of using bioactive agents to replace antibiotics to generate an environmentally friendly mode of culturing seahorses.

## Introduction

Seahorses (*Hippocampus* spp.) are highly specialized marine fishes that are often used as flagship species to gain support for marine conservation [1]. Seahorses are popular in traditional Chinese medicine (TCM) because they have high medicinal value, such as decreasing the hyperplasia of cancer cells, eliminating tumors, enhancing immunity, and strengthening kidneys [2]. Unique body morphology of seahorses, with their horse shaped head, male pregnancy, and vertical swimming, has also attracted much attention from aquarium hobbyists [3]. More than 25 million seahorses are traded annually for aquariums, as souvenirs, and as part of the TCM markets on a global scale [4].

However, wild seahorse populations have declined heavily in recent decades, due to over-fishing, pollution, and habitat loss [5]. Since 2004, all 33 recognized seahorse species have been included in Appendix II of CITES (Convention on International Trade in Endangered Species of Wild Fauna and Flora). In the last two decades, seahorse aquaculture has been proposed as a strategy to meet growing market demands [5] and to preserve the sustainability of wild populations [6].

Although there have been attempts to culture seahorses commercially for over 60 years [7], large-scale commercial production of many seahorse species remains challenging. The major bottlenecks for rearing seahorses include reproduction, juvenile survival, inadequate nutrition, and especially disease outbreak [8, 9]. Moreover, seahorses are sensitive to changes in the rearing environment of aquaculture systems, including water quality, water temperature [10], salinity, and light intensity[11].

Seahorses often acquire diseases during culturing, leading to high mortality rates in some farms [12]. The various pathogens of seahorse diseases include bacteria (e.g., *Vibrio* spp., *Mycobacterium hippocampi*) [5, 13–15], fungi [16], parasites (e.g., *Glugea heraldi*, *Philasterides dicentrarchi*, *Microsporidia* sp., *Amyloodinium ocellatum*) [15–18], and viruses [19]. To date, no effective measures exist to control most diseases of seahorses, except for bacterioses, which are primarily treated with antibiotics [5]. Various problems arise from the overuse of antibiotics, including the resistance of pathogens, environmental pollution of the culture conditions, and over standard residues that pollute the medicinal composition of seahorses [5].

Tail rot disease is associated with major economic losses in the seahorse aquaculture in China. Majority of seahorse farms in China are currently suffering from tail rot disease which caused by different pathogens [20, 21]. Qin et al. [20] reported that 85 seahorse farms in eight districts along the China’s coast were investigated to evaluate the epidemic situation of tail rot disease induced by *V*. *harveyi*. The results showed that tail rot disease was seriously inducing high mortality in most seahorse farms in China. The percentage of farms suffering serious tail rot disease ranged from 33.3% to 72.7% among eight different districts. We conducted seahorse disease investigations on several farms in Hainan province (China) since June, 2015. Almost all the farms investigated suffered from tail rot disease with mortalities reaching up to 15%, occasionally up to 40%. In general, one-month-old to two-month-old seahorse juveniles were prone to tail rot disease. It was found that tail rot disease causing the low survival rate of seahorse juveniles. Therefore, the prevention and control of this disease are essential for the development of seahorse aquaculture.

*Hippocampus kuda* is one of the 33 extant and endangered species, which is widely distributed throughout the tropical Indo-Pacific region, from the Indian subcontinent in the west to the Pacific islands in the east. It is one of the most heavily traded seahorse species [22]. Commercial seahorse culture of mainly *H*. *kuda* has been carried out in China for several years for Chinese traditional medicinal [23]. However, *H*. *kuda* are affected by frequent disease events, such as tail rot disease [24], deep skin ulcer disease [15], scuticociliatosis [17], haemorrhages disease [25], white patch disease [26], etc. This study aimed to isolate and identify the pathogenic agent that causes tail rot disease in cultured *H*. *kuda* from Hainan (China), and determine its antibiotic-resistance. Finally, antibacterial tests of the bioactive agents on the pathogen (in vitro) were conducted. Our results are expected to provide information to help reduce the use of antibiotics for treating tail rot disease, by suggesting a suitable biological method for the prophylaxis and treatment of seahorses.

## Materials and methods

### Experimental seahorses

Different batches of diseased and healthy cultured juvenile *H*. *kuda* were obtained from the seahorse breeding base of Longsheng Biotechnology Development Co., Ltd, Hainan, China (Lat 19°22′13.44″N; Long 110°39′57.40″E). The body height and the wet body weight of seahorses were measured by following the methods of Lourie et al. [22]. Ten diseased seahorses and 150 healthy seahorses were sampled. The size of the diseased individuals (about 8 weeks old) used to isolate the pathogens was wet body weight: 0.63 ± 0.09 g and body height: 4.2 ± 0.22 cm. The size of the healthy juvenile seahorses (about 5 weeks old) used for the artificial immersion test was wet body weight: 0.35 ± 0.02 g and body height: 3.01 ± 0.12 cm.

All healthy juvenile seahorses were temporarily cultured for one week in three tanks (100 cm × 48 cm × 40 cm), each with 50 individuals. Seawater in each culture tank was constantly aerated, with 20% of the seawater being exchanged daily. During the experiment, salinity, temperature, and light intensity were maintained at 35±1.0 ‰, 25±1.0°C, and 2500±200 lx, respectively. The seahorses were fed twice a day (09:00 and 16:00) with the sterile copepods. Feces were siphoned out of the tanks daily.

### Bioactive agents

Tea polyphenols, chitosan, chitin, betaine, kojic acid, gallic acid, nisin, allicin, alga polysaccharide, and polylysine were obtained from Xiya Chemical Co., Ltd (Shandong, China). Allicin, cranberry extract, echinacea extract, maca root powder, black cohosh extract, elderberry extract, ashwagandha extract, and rosemary leaf extract were purchased from Great Nature Care (GNC) Co., Ltd. (USA). These bioactive agents are routinely classified as antimicrobials on the basis of susceptibility tests that produce inhibitory concentrations in the range of 10 to 100 mg/L [27–29]. In this work, each product was tested at the concentration ranged from 5 to 100 mg/L.

### Isolation of bacteria

Tail rot disease is characterized by the occurrence of loss of prehensility in the tail, followed by whitening and tissue erosion starting at about 1 cm above the tip of the tail. Therefore, moribund *H*. *kuda* exhibiting these symptoms were collected. Ten morbid seahorse juveniles were anesthetized by clove oil (80 ppm, Hansi, China) immediately according to the methods reported by Rob et al. [30] and Leary et al. [31]. Then, they were sanitized three times with sterile saline, and were scrubbed with 75% (v/v) ethanol aseptically on a clean bench. Diseased seahorses were carefully dissected. Samples isolated from the liver, heart, kidney, spleen, intestine, stomach, lesion skin, and muscle were directly streaked on different plates containing Marine Agar 2216 (BD Difco) and were inoculated at 25°C for 7 days. The culturable strains were then subcultured on the same medium until pure isolates were consistently obtained. The pure isolates were stored at -80°C with 15% (v/v) glycerol until subsequent use.

### Identification of bacteria

Three culturable strains (HL11, HL12, and HL13) were isolated from the diseased *H*. *kuda* specimens. Strains were characterized based on its morphology, referring to Bergey’s Manual of Systematic Bacteriology [32]. PCR amplification of 16S rRNA sequences of bacterial isolates were conducted as described by the published procedures [33]. The *gyrB* gene sequence of strain HL11 was amplified through PCR with universal primers UP1 and UP2r applying the PCR conditions described [34]. Both 16S rRNA sequences and *gyrB* gene sequences were compared with the sequences in GenBank using BLASTN. Their phylogenetic analysis were carried out according to the published procedures [33, 34].

### Challenge experiments of the bacterial isolates

Three bacterial isolates that had been cultured at 25°C for 24 h were added to seawater to obtain final concentrations of approximately 5.0×10^7^ cfu/mL. Healthy seahorses were randomly selected, and assigned to three experimental treatments (HL11, HL12, and HL13) of eight individuals. These seahorses were immersed in seawater with strains for 12 h. Then, challenged seahorses were placed in a new tank (45 cm×48 cm×40 cm). All challenges were performed in duplicate, two control aquaria with eight control seahorses from the same source were exposed to the same amount bacteria-free seawater. During the challenge test, seahorses were fed and managed under the same rearing conditions as those in temporarily cultured tanks. Dead seahorses were removed immediately. During the 15-day experimental period, the mortality of seahorses was recorded daily. Then, re-isolation of different bacteria was performed on seahorses in the challenged groups.

### Antibiotic-resistance test

The antibiotic-resistance of strain HL11 was tested using the Kirby-Bauer disk diffusion method [35]. Thirty two antibiotics were chosen in accordance with the CLSI M100-S20 guidelines, and they represent antibiotics of clinical importance to *Pseudoalteromonas* spp. Strain HL11 was diluted to a final concentration of 10^8^ cfu/mL with sterile distilled water. A volume of 0.1 mL bacterial suspension was spread on the agar plates. The disks (6-mm diameter, Binhe, China) of different antibiotics were placed on the agar surface. The plates were inoculated at 30°C for 16–18 h, and the radius of the inhibition zone was determined to quantify the inhibition of bacterial growth. The sensitivity of the pathogen to antibiotics was classified as sensitive (S), intermediate (I), or resistant (R) according to the manufacturer’s standards.

### Antibacterial test in vitro

The susceptibilities of the pathogen to various bioactive agents were performed by following the agar diffusion-inhibition method with some modifications [36]. Strain HL11 was diluted to a final concentration of 10^8^ cfu/mL with sterile distilled water. A volume of 0.1 mL bacterial suspension was spread on the agar plates. Wells (6 mm in diameter) were punched into the agar plates, and four wells were pouched per plate. Then, 50 μL different concentrations of bioactive agents were placed in each hole and were incubated overnight at 30°C. The zone of bacterial growth inhibition was inspected and measured. Ampicillin and sterile distilled water were used as positive and negative controls, respectively. All tests were conducted in triplicate, and the results were presented as mean values.

### Ethics statement

All personnel involved with the care and use of seahorses must be adequately trained in basic principles of laboratory seahorses science, such as observing, recording, seawater exchanging, feeding etc., to help ensure high-quality science and seahorses well-being. All experimental procedures described in the present study were conducted in strict according to the recommendations in the Guide for the Care and Use of Laboratory Animals. The protocol was approved by the Institutional Animal Care and Use Committee of Hainan Tropical Ocean University (Protocol Number: 2016012). The study was approved by the Ethical Committee for Hainan Tropical Ocean University. All efforts were made to minimize suffering of the animals.

## Results

### Signs of tail rot disease

**Fig 1:**
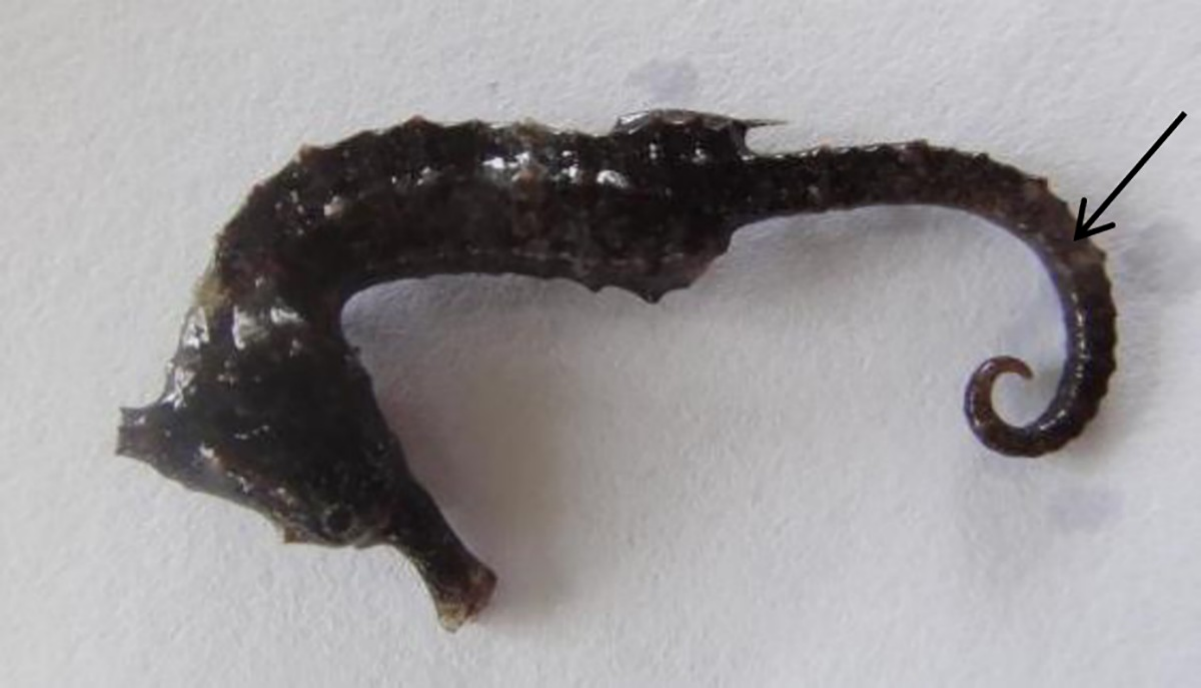
The clinical symptoms of *H*. *kuda* with tail rot disease. The arrow meant that grayish-white patches on the tail of seahorse.

Seahorses suffering from tail rot disease lost brightness on the body surface, became weak and anorexic. Diseased seahorses often floated in the water, swam slowly out of the group. The tail couldn’t be bent normally, and lost prehensility. Upon clinical examination of the affected seahorses, grayish-white patches on the tail and necrotic tail lesions were noted (Fig 1). Many of the infected seahorses stopped eating in severe cases, consequently resulting in the death. Pathological anatomy showed that intestine in diseased seahorses were transparent and swelling with yellow viscous liquid inside. Focally, necrotic muscle tissue was present underneath a necrotic epidermis in places where bacterial invasion was seen.

### Isolation and identification of bacteria

Three culturable strains (HL11, HL12, HL13) were isolated from the intestine, liver, and lesion skin in tail of the diseased *H*. *kuda* specimens, respectively. The isolates HL11 and HL13 were identified as Gram-negative bacteria, and the isolate HL12 was Gram-positive bacteria. The colonies of the bacterial isolates HL11, HL12, and HL13 on Marine Agar 2216 plate were light red, grayish white, and slightly cream in colour, respectively (S1 Fig). All strains were rod-shaped. Wherein, isolates HL12 and HL13 are motile by means of a flagellum. On the contrary, isolate HL11 didn’t have flagella, and were non-motile. The typical characteristics of three isolates included oxidase production and nitrate reduction, but not ornithine decarboxylase production, indole production, and malonate utilization, and so on. Other physiological and biochemical properties of three isolates are listed in Table 1.

**Table 1:** Physiological and biochemical properties of three isolates.

Characteristic	HL11	*spongiae*	HL12	*B*. *subtilis*	HL13	*P*. *ganghwense*
Motility	-	-	+	+	+	+
Oxidase	+	+	+	+	+	+
Ornithine decarboxylase	-	-	-	-	-	-
Lysine decarboxylase	+	-	-	-	-	+
Arginine dihydrolase	-	-	+	+	+	+
Urease	-	-	+	+	-	-
H_2_S production	+	+	-	-	-	-
Indole production	-	-	-	-	-	+
Citrate utilization	-	-	+	+	-	-
Malonate utilization	-	-	-	-	-	-
Hydrolysis of Gelatin	-	-	+	+	-	+
Nitrate reduction	+	+	+	+	+	+
Salicin	-	-	+	+	-	-
VP test	-	-	+	+	-	-
ONPG	-	-	-	-	-	-
Glucose	+	+	+	+	+	+
Mannitol	-	-	+	+	-	-
Xylose	-	-	+	+	-	-
Inositol	-	+	-	-	-	-
Sorbitol	-	-	+	+	-	-
Rhamnose	-	ND	-	ND	-	-
Sucrose	-	-	+	+	-	-
Lactose	-	-	+	+	-	-
Arabinose	-	-	+	+	-	-
Adonitol	-	ND	-	ND	-	ND
Raffinose	-	ND	-	+	-	ND

+, positive; -, negative; ND: data not described in Bergey's Manual of Systematic Bacteriology [32]; *P*. *spongiae*, *B*. *subtilis* and *P*. *ganghwense* type strains were compiled from Bergey' Manual [32].

**Fig 2:**
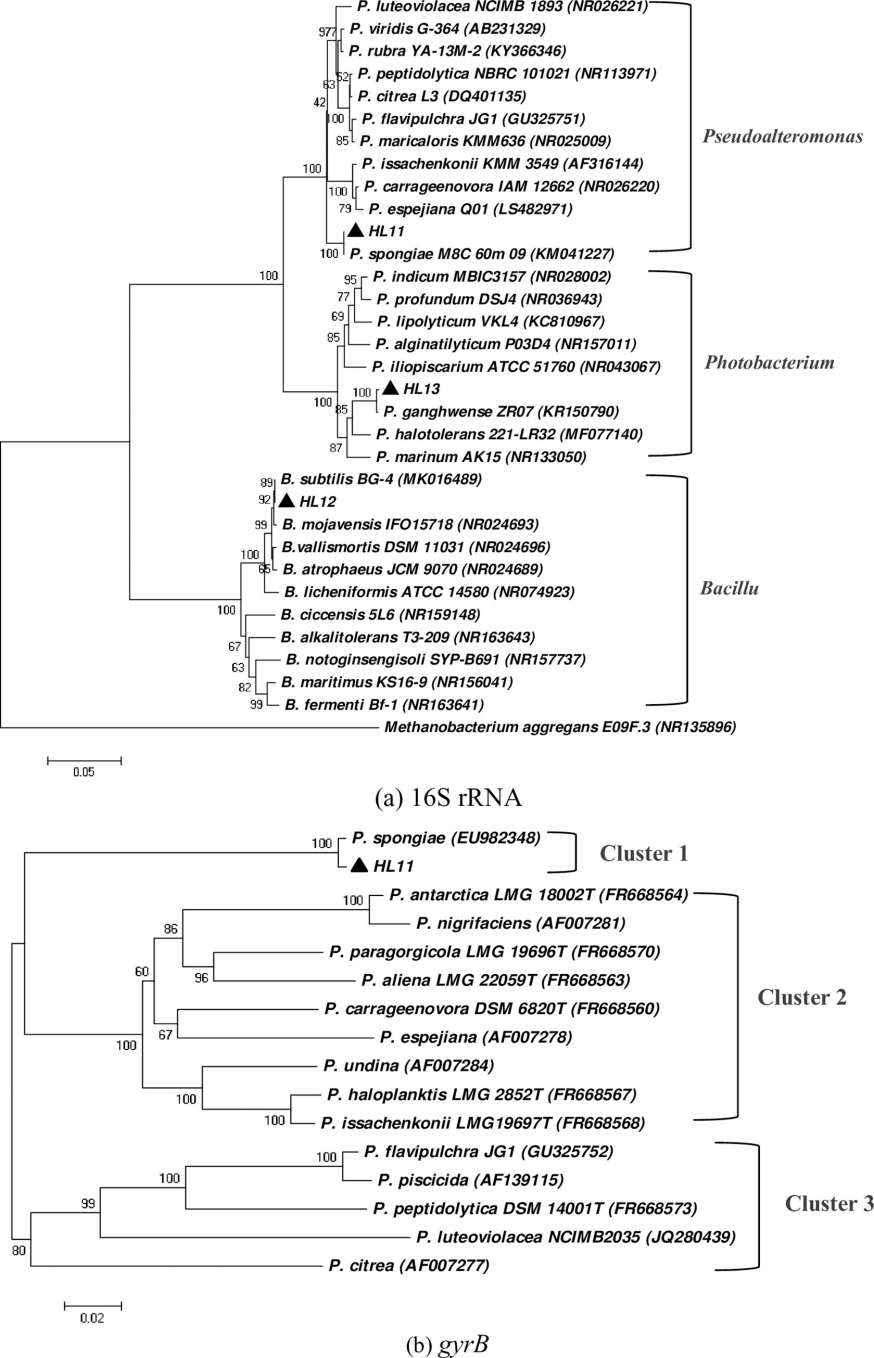
Phylogenetic trees based on 16S rRNA (a) and *gyrB* (b) sequences of different strains. The trees were constructed using the neighborjoining (NJ) algorithm in MEGA 7.0. Confidence for the tree topology was estimated using the bootstrap values based on 1000 replicates. Scale bar = 0.05 (or 0.02) substitutions per nucleotide position. The estimated genetic distance between sequences is proportional to the lengths of the horizontal lines connecting one sequence to another.

In order to determine the taxonomic positions of the bacteria, the sequences of 16S rRNA genes of the three isolates were amplified and compared with the relevant 16S rRNA sequence of bacteria in GenBank. The phylogenetic trees (Fig 2A) constructed using the 16S rRNA sequences of three isolates of bacteria and its close related species showed that the three isolates of bacteria clustered into three clades. Strain HL11 was identified as *Pseudoalteromonas spongiae* (GenBank accession no. MF774040) with high similarity (99.4%) to *P*. *spongiae* M8C_60m_09 (GenBank accession no. KM041227). The strain HL12 was identified as *Bacillus subtilis* (GenBank accession no. MK016489) with high sequence identity (99.8%), and strain HL13 was identified as *Photobacterium ganghwense* (GenBank accession no. KR150790) with high sequence identity (99.9%). The identification results were similar to those found through physiological and biochemical identification.

The more rapidly evolving *gyrB* gene (encode the subunit B protein of DNA gyrase) was utilized as a high-resolution molecular identification marker for discriminating strains of *Pseudoalteromonas* [37]. Sequence similarity analysis of the 1260 bp *gyrB* gene sequence of strain HL11 (Fig 2B) further confirmed that the strain belonged to the genus *Pseudoalteromonas*, showing high *gyrB* gene sequence similarities (99.3%) with *P*. *spongiae* (GenBank accession no. EU982348). The branching order of the *gyrB*-based tree (Fig 2B) resembled that of the tree derived from 16S rRNA nucleotide sequences (Fig 2A). Phylogenetic tree of *Pseudoalteromonas* clearly delineated three distinct clusters: cluster 1 contained *P*. *spongiae*; cluster 2 contained *P*. *citrea*, *P*. *flavipulchra*, *P*. *peptidolytica*, and *P*. *luteoviolacea*; cluster 3 contained *P*. *carrageenovora*, *P*. *espejiana*, and *P*. *issachenkonii*. Both 16S rRNA and *gyrB* sequences identified the isolate HL11 as a *P*. *spongiae* strain.

### Infection of pathogens

To confirm the pathogenicity and lethality of the three culturable strains, the isolates were used to infect healthy seahorses using the immersion-challenged method. In the HL11 infected groups, seahorse floated in the water, swam slowly at the early stage of infection, and then lied at the bottom of the tank. Before death, most seahorses exhibited: lack of appetite, erratic swimming behavior, portion of skin began to flake or lift up, accompanied by the presence of a ring of ulcers. As the disease progressed, the tip of the tail became white and the loss of coloration advanced further up the tail. Overall, the clinical signs were the same as those documented when natural tail rot disease is presented. During 15 days of the challenge experiments, death occurred from the fourth day post-challenge, with 100% mortality by day 13. In contrast, no mortality or visible disease symptoms were observed in the other two challenged groups (infected with strains HL12 or HL13) or the control groups.

Tissues of lesion skin in tail and enteric lysate were sampled from all diseased *H*. *kuda* specimens that developed symptoms in the challenged groups. Then, the pathogenic bacteria were re-isolated on Marine Agar 2216 plates. Many bacterial colonies with highly uniform morphological characteristics were isolated, which were identical to the colony morphology, the physiological and biochemical properties of *P*. *spongiae* HL11. The died seahorses in the challenged group were all positive for HL11, tested by inoculation on Marine Agar 2216. Attempts were made to isolate the pathogens from the seahorses in the other two challenged groups and the control groups at the same time as we isolated the pathogens from the diseased samples. *B*. *subtilis* and *P*. *ganghwense* were present in the seahorses groups that challenged by isolates HL12 and HL13, respectively. No other bacteria were isolated from the seahorses in the three challenged groups.

### Antibiotic-resistance test

The sensitivity of strain HL11 to 32 kinds of antibiotics was determined by the Kirby-Bauer disk diffusion method (presented in Table 2). The antibiotic-resistance test showed that this isolate was sensitive to 13 varieties of antibiotics, including ampicillin, aztreonam, ceftazidime, and so on. Among the others, it exhibited intermediate resistance to 6 kinds of antibiotics, including cefoxitin, cephalothin, ciprofloxacin, norfloxacin ofloxacin, norfloxacin. It was resistant to thirteen other types of antibiotics.

**Table 2:** Sensitivity of strain HL11 to various antibiotics.

Antibiotics	Concentration (μg/disk)	Sensitivity	Antibiotics	Concentration (μg/disk)	Sensitivity
Ampicillin	10	S	Ciprofloxacin	5	I
Aztreonam	30	S	Norfloxacin	10	I
Cefepime	30	S	Ofloxacin	5	I
Cefotaxime	30	S	Amikacin	30	R
Ceftazidime	30	S	Cefazolin	30	R
Ceftriaxone	30	S	Clarithromycin	15	R
Cefuroxime	30	S	Clindamycin	2	R
Chloromycetin	30	S	Cotrimoxazole	23.25	R
Levofloxacin	5	S	Erythromycin	15	R
Midecamycin	30	S	Gentamycin	10	R
Nitrofurantoin	300	S	Minocycline	30	R
Piperacillin	5	S	Oxacillin	1	R
Spectinomycin	100	S	Penicillin G	10	R
Cefoperazone	30	I	Tetracycline	30	R
Cefoxitin	30	I	Streptomycin	10	R
Cephalothin	30	I	Tobramycin	10	R

S, sensitive; I, intermediate; R, resistant.

### Antibacterial effect of bioactive agents to the pathogen

The antibacterial activity of the 18 kinds of bioactive agents against pathogen HL11 was determined by agar diffusion-inhibition method. The results (as shown in Table 3) suggested that the tested bacterium was susceptible to five bioactive agents, including tea polyphenols, lactic acid, gallic acid, polylysine, and allicin. No zone of inhibition was exhibited by the other 13 bioactive agents (nisin, chitosan, kojic acid, betaine, ginger extract, alga polysaccharide, cranberry extract, echinacea extract, maca root, black cohosh extract, elderberry extract, ashwagandha extract, rosemary leaves extract) (data not shown) and sterile distilled water. In general, the antibacterial activity of the different bioactive agents depended on their concentration and type. The antibacterial activity of five bioactive agents against strain HL11 increased with their dosage. Given the same concentration of various antibacterial agents, the mean diameters of the inhibition zones were ampicillin > polylysine > tea polyphenol > lactic acid> allicin.

**Table 3:** Antibacterial activity of different bioactive agents against the strain HL11.

Bioactive agents	Concentration (mg/L)	Radius of the inhibition zone (mm)	Bioactive agents	Concentration (mg/L)	Radius of the inhibition zone (mm)
Tea polyphenol	100	20.5±1	Polylysine	100	21.0±1
	50	17.0±1		50	16.0±1
	25	14.5±1		25	14.5±1
	12.5	12±0.5		12.5	13±0.5
Lactic acid	100	20.0±1	Allicin	100	18.0±1
	50	16.5±1		50	14.0±1
	25	14.5±1		25	12.5±1
	12.5	11±0.5		12.5	10.5±0.5
Gallic acid	30	19.0±1	Ampicillin	100	22.0±1
	15	15.0±1		50	18.0±1
	10	13.5±1		25	15±1
	5	0		12.5	13.5±1
Sterile distilled water	0	0			

Values are means ± SD resulting from at least three independent experiments performed in triplicate.

## Discussion

In this study, three culturable bacteria (HL11, HL12, HL13) were isolated from the intestine, liver, and lesion skin in tail of seahorses with tail rot disease, respectively. Based on the morphological characteristics, physiological and biochemical properties, 16S rRNA sequences, the strain HL11, HL12, and HL13 were identified as *P*. *spongiae*, *B*. *subtilis* and *P*. *ganghwense*, respectively. In addition, the strain HL11 was also identified as *P*. *spongiae* by *gyrB* gene sequences. Challenge experiments and bacterial re-isolation experiments identified the isolate HL11 as the causative pathogen of the disease. Healthy *H*. *kuda* that was experimentally challenged with the isolate *P*. *spongiae* HL11 exhibited the same clinical signs as observed in naturally infected cases.

Several pathogens are responsible for tail rot disease in different seahorses. *Vibrio splendidus* (*H*. *hippocampus*) [38], *V*. *alginolyticus* [38] and *Mycobacterium hippocampi* (*H*. *guttulatus*) [13], *Tenacibaculum aestuarii* (*H*. *kuda*) [24], *V*. *harveyi* [20] and *V*. *rotiferianus* (*H*. *erectus*) [21] have been identified as the causative agents of tail rot disease in seahorses. The genus *Pseudoalteromonas* has been reported to produce biologically active compounds with anti-fouling, antimicrobial, algicidal, and various pharmaceutically relevant activities [39–41]. However, only few of *Pseudoalteromonas* are related to pathologies of some mariculture animals [42–44]. The present study demonstrated that *P*. *spongiae* can directly and seriously destroy the seahorse intestine and skin in tail, so once the cultured seahorses were infected by *P*. *spongiae*, they will die sooner or later. Although being naturally occurring bacteria and ubiquitous members of the marine environment, reports on *P*. *spongiae* as pathology in seahorses is very scarce. To the best of our knowledge, this study is the first to describe *P*. *spongiae* as the causative agent of tail rot disease in *H*. *kuda*.

The pathogen *P*. *spongiae* HL11 was sensitive to 13 kinds of 32 tested antibiotics, including ampicillin, ceftazidime, aztreonam, and so on. According to the guidelines for the use of antibiotics in Chinese aquaculture [45], wherein chloromycetin was not authorized, and the other 12 susceptible antibiotics of *P*. *sponge* HL11 were neither in authorized list nor in banned list. Therefore, these 13 susceptible antibiotics were currently not recommended for use in seahorses aquaculture. On the other hand, *P*. *sponge* exhibited high resistance to the other 13 antibiotics, such as tetracycline, penicillin G, erythromycin, streptomycin, etc. It may be attributed to the excessive and inadequate use of antibiotics in aquaculture, and transmission of resistance within and between bacteria. Both oxytetracycline and povidone iodine have been used to control the tail rot disease of cultured seahorses. However, these agents often fail to control the disease when serious clinical symptoms are presented. Unsuccessful attempts were also made to control tail rot disease in *H*. *guttulatus* by topical treatments (formalin + malachite green, antibiotics, iodine or fresh-water baths) [46]. Besides, antibiotics used for treating infectious diseases of seahorses have caused various problems [5]. Hence, it is exigent to develop a novel, environment friendly approach which could be an alternative to antibiotics.

Natural plants (especially medicinal plants) and their effective components exhibit remarkable antibacterial activity and have become the hotspot of aquatic drugs. Consequently, this study investigated the antibacterial activity of different bioactive agents on the pathogen HL11. Five bioactive agents (including tea polyphenols, lactic acid, gallic acid, polylysine, and allicin) were found to inhibit the growth of the pathogen. At the same concentration, the mean radius of the inhibition zone of the five bioactive agents to the pathogen was comparable to that of the most frequently used antimicrobial drugs (eg. enrofloxacin, florfenicol, neomycin sulfate) (data not shown). Cell membrane damage was assumed to be the main mechanism of tea polyphenols, lactic acid, gallic acid, and polylysine involved in the antibiotic effect toward *P*. *sponge* HL11. They could led to irreversible changes in membrane properties (charge, intra and extracellular permeability, and physicochemical properties) through hydrophobicity changes, decrease of negative surface charge, and occurrence of local rupture or pore formation in the cell membranes with consequent leakage of essential intracellular constituents. The same phenomenon was observed in bacteria such as *Escherichia coli*, *Pseudomonas aeruginosa*, *Staphylococcus aureus*, etc. [21–22, 27]. The main antimicrobial effect of allicin may be due to its interaction with important thiol-containing enzymes which are critical for maintaining the correct redox state within bacteria [47]. However, further research is imperative because the inhibition mechanism of these bioactive agents against *P*. *sponge* HL11 are merely speculative.

As natural food and medicine materials, these bioactive agents have many merits. Such merits include no drug residues and resistance, slight toxic and side effects, no harm to health, high efficiency, and no pollution of the surrounding aquaculture environment. The results of the present work indicated the potential of using these bioactive agents to replace the antibiotics for safe use on seahorses. This study provided baseline information towards developing an environmentally friendly culture mode (non-toxic side effects, no drug residues) for rearing healthy seahorses under aquaculture conditions.

## Conclusion

In this study, *P*. *spongiae* HL11 was isolated and identified as the pathogenic bacteria causing tail rot disease in common seahorse (*H*. *kuda*). Five bioactive agents that inhibited the growth of the pathogen HL11 were screened out. In the future studies, statistical optimization of the five bioactive agents for seahorse aquaculture, the antibacterial mechanism of these bioactive agents, and how them interact with one another, are the themes of ongoing projects.

## Supporting information

S1 Fig Colonies of three isolates on Marine Agar 2216 plate. (DOCX)

## References

[1] Koldewey HJ, Martin-Smith KM. A global review of seahorse aquaculture. Aquaculture. 2010; 302(3): 131–152. 10.1016/j.aquaculture.2009.11.010

[2] Zhang CH, Xu GJ, Xu LS, Wang Q. Physical and chemical analysis of medicinal animals of Syngnathidae. Chinese Medicine. 1997; 20: 140–144 (in Chinese). 10.13863/j.issn1001-4454.1997.03.012 12572445

[3] Luo W, Wang X, Qu HY, Qin G, Zhang HX, Lin Q. Genomic structure and expression pattern of MHC II α and II β genes reveal an unusual immune trait in lined seahorse *Hippocampus erectus*. Fish Shellfish Immun. 2016; 58: 521–529. 10.1016/j.fsi.2016.09.057 27697560

[4] Salin KR, Mohankumaran NC. Resources and biodiversity of seahorses and the need for their conservation in India. Aquaculture Asia. 2006; 10: 3–8.

[5] Lin TT, Zhang D, Liu X, Xiao DX. Variations of immune parameters in the lined seahorse *Hippocampus erectus* after infection with enteritis pathogen of *Vibrio parahaemolyticus*. Fish Shellfish Immun. 2016; 50: 247–254. 10.1016/j.fsi.2016.01.039 26851568

[6] Faleiro F, Narciso L. Prey-predator dynamics in seahorses (*Hippocampus guttulatus*): deciphering fatty acid clues. Aquac Res. 2013; 44(4): 618–633. 10.1111/j.1365-2109.2011.03067.x

[7] Zou ZH. Rearing seahorse. China Fisheries. 1958: 13 (in Chinese).

[8] Zhang D, Yin F, Lin JD. Criteria for assessing juvenile quality of the lined seahorse, *Hippocampus erectus*. Aquaculture. 2011; 322–323: 255–258. 10.1016/j.aquaculture.2011.10.008

[9] Ko J, Wan Q, Bathige SDNK, Lee J. Molecular characterization, transcriptional profiling, and antibacterial potential of G-type lysozyme from seahorse (*Hippocampus abdominalis*). Fish Shellfish Immun. 2016; 58: 622–630. 10.1016/j.fsi.2016.10.014 27732899

[10] Aurélio ML, Faleiro F, Pimentel MS, Narciso L, Rosa R. Physiological and behavioral responses of temperate seahorses (*Hippocampus guttulatus*) to environmental warming. Mar Biol. 2013; 160(10): 2663–2670. 10.1007/s00227-013-2259-8

[11] Lin Q, Zhang D, Lin JD. Effects of light intensity, stocking density, feeding frequency and salinity on the growth of sub-adult seahorses *Hippocampus erectus* Perry, 1810. Aquaculture. 2009; 292: 111–116. 10.1016/j.aquaculture.2009.03.028

[12] Lin Q, Lin J, Huang L. Effects of light intensity, stocking density and temperature on the air-bubble disease, growth and survivorship of early juvenile seahorse *Hippocampus erectus* Perry, 1810. Aquac Res. 2010; 42(1): 91–98. 10.1111/j.1365-2109.2010.02573.x

[13] Balcázar JL, Planas M, Pintado J. *Mycobacterium hippocampi* sp. nov., a rapidly growing scotochromogenic species isolated from a seahorse with tail rot. Curr Microbiol. 2014; 69(3): 329–333. 10.1007/s00284-014-0588-6 24801334

[14] Binh DT, Quyen VHQ, Sang TQ, Oanh TT. Vibriosis in cultured seahorse (*Hippocampus spp*.) in Khanh Hoa Province, Vietnam. International Journal of Innovative Studies in Aquatic Biology and Fisheries (IJISABF). 2016; 2: 43–50.

[15] Sanaye SV, Pawar HB, Murugan A, Sreepada RA, Singh T, Ansari ZA. Diseases and parasites in cultured yellow seahorse, *Hippocampus kuda* (Bleeker, 1852). Fish Chimes. 2013; 32 (11): 65–67. 10.1017/S003871340001959X

[16] Koldewey H. Seahorse Husbandry in Public Aquariums: Manual with chapters contributed by members of the Syngnathid Discussion Group. Zoological Society of London, London, 2005.

[17] Vincent ACJ, Clifton-Hadley RS. Parasitic Infection of the Seahorse (*Hippocampus erectus*)—A Case Report. J Wildlife Dis. 1989; 25(3): 404–406. 10.7589/0090-3558-25.3.404 2761014

[18] Shin SP, Han JE, Gomez DK, Kim JH, Choresca CHJ, Jun JW, et al Identification of scuticociliate *Philasterides dicentrarchi* from indo-pacific seahorses *Hippocampus kuda*. Afr J Microbiol Res. 2011; 5(7): 738–741. 10.5897/AJMR10.294

[19] Mundell NA, Beier KT, Pan YA, Lapan SW, Ayturk DG, Berezovskii VK, et al Vesicular stomatitis virus enables gene transfer and transsynaptic tracing in a wide range of organisms. J Comp Neurol. 2015; 523(11): 1639–1663. 10.1002/cne.23761 25688551 PMC4458151

[20] Qin G, Wang X, Tan S, Lin Q. A bacterial infection by *Vibrio harveyi* causing heavy reduction of cultured lined seahorse *Hippocampus* erectus. J Fish Dis. 2017; 40: 601–605. 10.1111/jfd.12533 27553601

[21] Yang QH, Zheng LY, Huang ZC, Lin Q, Lu Z, Zhou C. Identification and characterization of pathogen *Vibrio rotiferianus*, a pathogen isolated from *Hippocampus erectus* with tail-rot disease. Journal of Fishery Sciences of China. 2017; 24(05): 1131–1140 (in Chinese). 10.3724/SP.J.1118.2017.16310

[22] Lourie SA, Stanley HF, Vincent ACJ, Hall HJ, Pritchard JC, Casey SP. Seahorse: An Identification Guide to the World's Species and their Conservation. Project Seahorse London, 1999, pp.214.

[23] Lin Q. Lin JD, Zhang D. Breeding and juvenile culture of the lined seahorse, *Hippocampus erectus* Perry, 1810. Aquaculture. 2008; 277: 287–292. 10.1016/j.aquaculture.2008.02.030

[24] Declercq AM, Chiers K, Broeck WVD, Rekecki A, Teerlinck S, Adriaens D, et al White necrotic tail tips in estuary seahorses, *Hippocampus kuda*, Bleeker. J Fish Dis. 2013; 37(5):501–504. 10.1111/jfd.12138 23763536

[25] Tendencia EA. The first report of *Vibrio harveyi* infection in the seahorse *Hippocampus kuda* Bleekers 1852 in the Philippines. Aquac Res. 2004; 35: 1292–1294. 10.1111/j.1365-2109.2004.01109.x

[26] Thampi Raj S, Lipton AP, Chauhan GS. Characterization and infectivity evaluation of *Vibrio harveyi* causing white patch disease among captive reared seahorses, *Hippocampus kuda*. Indian J Mar Sci. 2010; 39(1): 151–156. 10.1093/icesjms/fsp239

[27] Bansal S, Choudhary S, Sharma M, Kumar SS, Lohan S, Bhardwaj V, et al Tea: A native source of antimicrobial agents. Food Res Int. 2013; 53: 568–584. 10.1016/j.foodres.2013.01.032

[28] Dai XM, An JX, Wang YN, Wu ZM, Zhao Y, Guo QQ, et al Antibacterial amphiphiles based on ε-polylysine: synthesis, mechanism of action, and cytotoxicity. RSC Adv. 2015; 5: 69325–69333. 10.1039/c5ra10393b

[29] Wang CJ, Chang T, Yang H, Cui M. Antibacterial mechanism of lactic acid on physiological and morphological properties of *Salmonella Enteritidis*, *Escherichia coli* and *Listeria monocytogenes*. Food Control. 2015; 47: 231–236. 10.1016/j.foodcont.2014.06.034

[30] Rob J, Jon D. Humane Euthanasia Techniques for Ornamental Fish. Pet Industry Association of Australia, Australia. http://piaa.net.au/wp-content/uploads/2015/10/Humane-Euthanasia-Techniques-for-Ornamental-Fish-AAWS-Document.pdf

[31] Leary S, Underwood W, Anthony R, Cartner S, Douglas Corey, Grandin T, et al AVMA Guidelines for the Euthanasia of Animals: 2013 Edition. American Veterinary Medical Association, Schaumberg IL, USA, 2013.

[32] Brenner DJ, Krieg NR, Staley JT. Bergey's Manual of Systematic Bacteriology, second ed vol. 2 Springer, New York, 2008.

[33] Zheng YF, Yu M, Liu Y, Su Y, Xu T, Yu MC, et al Comparison of cultivable bacterial communities associated with Pacific white shrimp (*Litopenaeus vannamei*) larvae at different health statuses and growth stages. Aquaculture. 2016; 451: 163–169. 10.1016/j.aquaculture.2015.09.020

[34] Yamamoto S, Harayama S. PCR amplification and direct sequencing of *gyrB* genes with universal primers and their application to the detection and taxonomic analysis of *Pseudomonas putidastrains*. Appl Environ Microbiol. 1995; 61: 1104–1109. 10.1016/j.ijms.2010.08.029 7793912 PMC167365

[35] Bauer AW, Kirby WM, Sherris JC, Turck M. Antibiotic susceptibility testing by a standardized single disk method. Am J Clin Pathol. 1966; 45: 493–496. 10.1093/ajcp/45.4_ts.493 5325707

[36] Rezk A, Nolzen J, Schepker H, Albach DC, Brix K, Ullrich MS. Phylogenetic spectrum and analysis of antibacterial activities of leaf extracts from plants of the genus *Rhododendron*. BMC Complem Alt M. 2015; 15: 67–76. 10.1186/s12906-015-0596-5 PMC4367927 25879877

[37] Zeng YX, Zheng TL. Relationships between two *Pseudoalteromonas* strains isolated from the Canada Basin and the Southern Ocean using a polyphasic approach. Adv Polar Sci. 2011; 22(1):25–34. 10.3724/SP.J.1085.2011.00025

[38] Balcázar JL, Gallo-Bueno A, Planas M., Pintado J. Isolation of *Vibrio alginolyticus* and *Vibrio splendidus* from captive-bred seahorses with disease symptoms. Anton Leeuw. 2010; 97(2): 207–210. 10.1007/s10482-009-9398-4 19921544

[39] Egan S, Holmström C, Kjelleberg S. *Pseudoalteromonas ulvae* sp. nov., a bacterium with antifouling activities isolated from surface of a marine alga. Int J Syst Evol Micr. 2001; 51: 1499–1504. 10.1099/00207713-51-4-1499 11491351

[40] Nikolaj GV, Månsson M, Nielsen FK. Bioactivity, Chemical Profiling, and 16S rRNA-Based Phylogeny of *Pseudoalteromonas* Strains Collected on a Global Research Cruise. Mar Biotechnol. 2011; 13(6): 1062–1073. 10.1007/s10126-011-9369-4 21305330

[41] Sun X, Zhao GM, Gu CM, Liu L, Zhu M, Liu ZP. Screening for and identification of an anti-clam *Vibrio* marine bacterium from an aquaculture pond in the Yellow Sea. Clean-Soil, Air, Water. 2016; 44(3): 304–308. 10.1002/clen.201200478

[42] Pujalte MJ, Sitjà-Bobadilla A, Macián MC, ÁLlvarez-Pellitero P, Garay E. Occurrence and virulence of *Pseudoalteromonas* spp. in cultured gilthead sea bream (*Sparus aurata* L.) and European sea bass (*Dicentrarchus labrax* L.). Molecular and phenotypic characterisation of *P*. *undinastrain* U58. Aquaculture. 2007; 271 (1): 47–53. 10.1016/j.aquaculture.2007.06.015

[43] Beurmann S, Ushijima B, Videau P, Svoboda CM, Smith AM, Rivers OS, et al *Pseudoalteromonas piratica* strain OCN003 is a coral pathogen that causes a switch from chronic to acute *Montipora* white syndrome in *Montipora capitata*. PLOS ONE. 2017, 12(11): e0188319 10.1371/journal.pone.0188319 29145488 PMC5690655

[44] Zheng YF, Yu M, Liu Y, Su Y, Xu T, Yu MC, et al Comparison of cultivable bacterial communities associated with Pacific white shrimp (*Litopenaeus vannamei*) larvae at different health statuses and growth stages. Aquaculture. 2016; 451: 163–169. 10.1016/j.aquaculture.2015.09.020

[45] Committee of Chinese Veterinary Pharmacopoeia. Chinese Veterinary Pharmacopoeia (2010 Edition). China Agriculture Press, Beijing, China 2011.

[46] Planasa M, Chamorro A, Quintas P, Vilar A. Establishment and maintenance of threatened long-snouted seahorse, *Hippocampus guttulatus*, broodstock in captivity. Aquaculture. 2008; 283: 19–28. 10.1016/j.aquaculture.2008.06.023

[47] Ankri S, Mirelman D. Antimicrobial properties of allicin from garlic. Microbes Infect, 1999; 2: 125−129. 10.1016/s1286-4579(99)80003-3 10594976

