# Supplementary figures and images for: Isolation, identification, and biological control in vitro of tail rot pathogen strain from Hippocampus kuda
Source: PLoS One. 2020 Apr 24;15(4):e0232162. doi: 10.1371/journal.pone.0232162 (PMC7182253; doi:10.1371/journal.pone.0232162)

Supplemental Fig. 1 Colonies of three isolates on Marine Agar 2216 plate

| 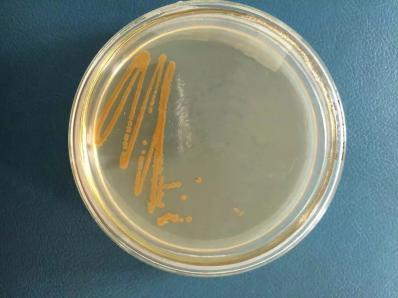 | 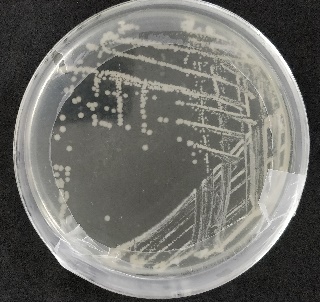 | 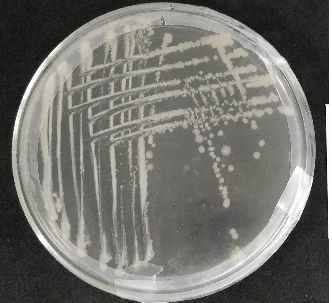 |
| --- | --- | --- |
| HL11 | HL12 | HL13 |
| Supplemental Fig. 1 | | |

Supplement: S1 Fig — (DOCX) [file pone.0232162.s001.docx]
